# Prognostic value of soluble ST2, high-sensitivity cardiac troponin, and NT-proBNP in type 2 diabetes: a 15-year retrospective study

**DOI:** 10.1186/s12933-022-01616-3

**Published:** 2022-09-10

**Authors:** Jacopo Sabbatinelli, Angelica Giuliani, Anna Rita Bonfigli, Deborah Ramini, Giulia Matacchione, Carla Campolucci, Artan Ceka, Elena Tortato, Maria Rita Rippo, Antonio Domenico Procopio, Marco Moretti, Fabiola Olivieri

**Affiliations:** 1 Department of Clinical and Molecular Sciences, Università Politecnica Delle Marche, Via Tronto 10/A, 60126 Ancona, Italy; 2 Laboratory Medicine Unit, Azienda Ospedaliero Universitaria “Ospedali Riuniti”, Via Conca 71, 60126 Ancona, Italy; 3 Scientific Direction, IRCCS INRCA, Ancona, Italy; 4 Clinic of Laboratory and Precision Medicine, IRCCS INRCA, Ancona, Italy; 5 Metabolic Diseases and Diabetology Department, IRCCS INRCA, Ancona, Italy

**Keywords:** Type 2 diabetes, Troponin, Natriuretic peptides, Soluble ST2, Cardiovascular risk

## Abstract

**Background:**

Patients with type 2 diabetes (T2DM) present an increased risk of cardiovascular (CV) disease and excess CV-related mortality. Beyond the established role of brain natriuretic peptide (BNP) and cardiac troponins (cTn), other non-cardiac-specific biomarkers are emerging as predictors of CV outcomes in T2DM.

**Methods:**

Serum levels of soluble suppression of tumorigenesis 2 (sST2), high-sensitivity (hs)-cTnI, and N-terminal (NT)-proBNP were assessed in 568 patients with T2DM and 115 healthy controls (CTR). Their association with all-cause mortality and the development of diabetic complications was tested in T2DM patients over a median follow-up of 16.8 years using Cox models and logistic regressions.

**Results:**

sST2 followed an increasing trend from CTR to uncomplicated T2DM patients (T2DM-NC) to patients with at least one complication (T2DM-C), while hs-cTnI was significantly higher in T2DM-C compared to CTR but not to T2DM-NC. A graded association was found between sST2 (HR 2.76 [95% CI 1.20–6.33] for ≥ 32.0 ng/mL and 2.00 [1.02–3.94] for 16.5–32.0 ng/mL compared to < 16.5 ng/mL, C-statistic = 0.729), NT-proBNP (HR 2.04 [1.90–4.55] for ≥ 337 ng/L and 1.48 [1.05–2.10] for 89–337 ng/L compared to < 89 ng/L, C-statistic = 0.741), and 15-year mortality in T2DM, whereas increased mortality was observed in patients with hs-cTnI ≥ 7.8 ng/L (HR 1.63 [1.01–2.62]). A ‘cardiac score’ based on the combination of sST2, hs-cTnI, and NT-proBNP was significantly associated with all-cause mortality (HR 1.35 [1.19–1.53], C-statistic = 0.739) and development of CV events.

**Conclusions:**

sST2, hs-cTnI, and NT-proBNP are associated with 15-year mortality and onset of CV events in T2DM. The long-term prognostic value of sST2 and its ability to track variables related to insulin resistance and associated metabolic disorders support its implementation into routine clinical practice.

**Supplementary Information:**

The online version contains supplementary material available at 10.1186/s12933-022-01616-3.

## Background

Current epidemiologic data have demonstrated that type 2 diabetes (T2DM) is accompanied by a 2–4-fold greater overall risk of cardiovascular (CV) complications compared to non-diabetic patients, even after adjustment for traditional risk factors [1]. Risk models for use in individuals with diabetes typically do not include information from biomarkers other than cholesterol, glycated hemoglobin (HbA1c), and urinary albumin-to-creatinine ratio (UACR), raising the question of whether adding novel biomarkers would improve CV risk prediction in patients with T2DM [2].

A large body of evidence was published on the associations of single biomarkers with CV risk in T2DM, but simultaneous evaluations of large numbers of biomarkers in diabetic populations have been limited [3–5]. More recently, a role for cardiac biomarkers has been advocated in the setting of CV outcome trials in T2DM [6, 7]. While their assessment has been shown to invariably improve the discrimination and reclassification of the risk of developing endpoints such as major adverse CV events (MACE) [8, 9], there is still uncertainty on whether their serial measurement may be useful to predict CV mortality or the cardioprotective benefits of T2DM medications [10–12]. Though the cardiac-specific biomarkers brain natriuretic peptide (BNP) and cardiac troponins (cTn) are firmly established as cornerstones of the diagnosis of heart failure (HF) and myocardial injury, respectively [13], a growing body of evidence supported the role of several non-cardiac specific biomarkers in describing the most relevant pathophysiological features of HF, i.e., inflammation, oxidative stress, extracellular matrix remodeling, neurohormonal activation, myocyte injury and stress [14, 15]. Among them, soluble suppression of tumorigenesis-2 (sST2), a member of the interleukin 1 receptor family, emerged as an efficient prognostic biomarker for patients with chronic HF [16]. sST2 is mainly produced in extracardiac tissues in response to proinflammatory and profibrotic stimuli [17]. By acting as a circulating decoy for interleukin-33 (IL-33), it prevents the cardioprotective effects of the ST2/IL-33 signaling, thus promoting maladaptive myocardial hypertrophy and cardiomyocyte apoptosis [18]. While a number of observational studies have investigated sST2 levels in T2DM [19, 20], data on its prognostic role is limited to short follow-up periods, and its incremental value over the established cardiac biomarkers—BNP and cTn–has not been extensively assessed.

The main objective of the present study was to investigate the association of high-sensitivity (hs)-cTnI, N-terminal (NT)-proBNP, and sST2, alone or in combination, with all-cause mortality and development of diabetic complications, including MACE, in patients with T2DM. Moreover, we aimed at comparing the levels of these biomarkers between patients with T2DM and healthy control subjects and assessing their correlations with variables assessing blood glucose control, organ damage, and T2DM clinical features.

## Materials and methods

### Study population

Samples were retrieved from a previously published cohort composed of 568 patients with T2DM and 618 presumably healthy controls [21]. Patients and controls were recruited at the Metabolic Diseases and Diabetology Department of IRCCS INRCA between May 2003 and November 2006. All T2DM patients (median age = 67 years, IQR 61–72 years.) and 115 randomly selected age- and sex-matched presumably healthy control subjects (CTR) (median age = 68 years, IQR 62–73 years) were included in the present investigation. T2DM was diagnosed according to the ADA criteria, i.e., patients having an HbA1C ≥ 6.5% or fasting blood glucose ≥ 126 mg/dl or 2 h blood glucose levels ≥ 200 mg/dl after OGTT, or a random blood glucose ≥ 200 mg/dl when severe diabetes symptoms are present [22]. Inclusion criteria for patients with diabetes were BMI < 40 kg/m^2^, age 40–87 years, ability and willingness to give written informed consent.

Subjects enrolled in the CTR group underwent a medical evaluation that included a complete history and clinical examination. Subjects with a clinical history of coronary artery disease, stroke, and peripheral vascular diseases were excluded from the study. Hypertension was defined as a systolic blood pressure > 140 mm Hg and/or a diastolic blood pressure > 90 mm Hg. Subjects with either a positive history of diabetes mellitus or a fasting blood glucose ≥ 126 mg/dL after confirmation on repeat testing were considered diabetic and excluded. Fasting blood samples of all subjects were processed to obtain serum and stored at − 80 °C.

The study was approved by the Institutional Review Board of IRCCS INRCA hospital (Approval No. 34/CdB/03). Written informed consent was obtained from each participant in accordance with the principles of the Declaration of Helsinki.

### Biomarkers

All serum samples were screened for hemolysis prior to analysis. sST2 serum levels were assessed on an Atellica^®^ CH 930 Analyzer (Siemens Healthineers, Germany) using the latex bead turbidimetric immunoassay Sequent-IA^™^ ST2 Assay (Critical Diagnostics, United States), currently available in Europe under the CE-IVD mark and intended to be submitted for US FDA review. The assay uses the same pair of monoclonal antibodies that are used in the FDA cleared Presage^®^ ST2 Assay (Critical Diagnostics). The linearity range is 15–300 ng/mL, the intra- and inter-assay CVs are 6.5% and 8.6%, respectively, at a sST2 concentration of 26 ng/mL. At a sST2 concentration of 75 ng/mL, the CV values are 2.8% and 3.8% respectively. Serum hs-cTnI and NT-proBNP were assessed on a Dimension Vista^®^ 1500 automated chemistry analyzer using the Dimension Vista^®^ high-sensitivity troponin I (TNIH) and NT-proBNP (PBNP) assays (Siemens Healthineers, Germany) based on LOCI^®^ technology. For the TNIH assay, the Limit of Blank (LoB), Limit of Detection (LoD), and Limit of Quantitation (LoQ) are 1.0 ng/L, 2.0 ng/L, and 3.0 ng/L, respectively. The linearity range reported by the manufacturer is 3.0–25,000.0 ng/L. 99th percentiles determined for serum are 51.1 ng/L for females and 74.9 ng/L for males. For the PBNP assay, the LoB, LoD, and LoQ are 0.2 ng/L, 0.8 ng/L, and 5.0 ng/L. The linearity range reported by the manufacturer is 5.0–35,000.0 ng/L. For the comparison of hs-cTnI methods, a subgroup of serum samples was assessed on an Architect i1000SR Immunoassay Analyzer using the ARCHITECT STAT High Sensitivity Troponin-I assay (Abbott Diagnostics, Illinois, USA). The LoB, LoD, and LoQ are 0.7–1.3 ng/L, 1.1–1.9 ng/L, and 1.5–2.9 ng/L, respectively. The linearity range reported by the manufacturer is 3.0–25,000.0 ng/L. 99th percentiles determined for lithium heparin plasma are 53.7 ng/L for females and 78.5 ng/L for males. The following cut-off points are recommended by the manufacturer for stratifying the risk of CV disease in asymptomatic individuals: males, low, < 6 ng/mL, moderate, 6–12 ng/mL, high > 12 ng/mL; females, low, < 4 ng/mL, moderate, 4–10 ng/mL, high > 10 ng/mL.

### Outcomes

Outcome events were measured as new onset of MACE (in patients without a history of MACE at the time of enrolment), T2DM complications (in patients without a history of T2DM complications at the time of enrolment), and all-cause mortality. MACE was defined as the nonfatal occurrence of myocardial infarction, cardiac arrest, cardiogenic shock, life-threatening arrhythmia, or stroke. Moreover, a composite endpoint of MACE and all-cause mortality was assessed. Follow-up information on outcomes was collected from medical records from the date of enrollment (May 2003–November 2006) to the last day of follow-up (31st December 2019). Specifically, follow-up information on BMI, blood glucose control (HbA1c), renal function (eGFR, urinary albumin-to-creatinine ratio), and development of T2DM complications, including MACE, was collected.

### Covariates

Information on vital signs, anthropometric data, medical history, behaviors, exercise, and concomitant treatments was collected for all the participants. Blood cell count and biochemical variables were assessed by standard procedures in all subjects. The estimated glomerular filtration rate (eGFR) was calculated according to CKD-EPI (Chronic Kidney Disease Epidemiology Collaboration) equation based on serum creatinine, age, sex, and ethnicity. The presence of diabetic complications was established as previously described [23]. Diabetic retinopathy was assessed by fundoscopy through dilated pupils and/or fluorescence angiography; incipient nephropathy was defined as a urinary albumin excretion rate > 30 mg/24 h and a normal creatinine clearance; neuropathy was established by electromyography; ischemic heart disease was defined by clinical history and/or ischemic electrocardiographic alterations; peripheral artery disease, including atherosclerosis obliterans and cerebrovascular disease based on history, was defined with physical examinations and Doppler velocimetry. At baseline, 103 patients were affected by neuropathy, 53 by atherosclerotic vascular disease, 84 by major adverse cardiovascular events (MACE), 74 by nephropathy, and 156 by retinopathy.

### Statistical analysis

Continuous variables were reported as either mean and standard deviation or median and interquartile range based on their distribution (assessed using Shapiro–Wilk test). For the analysis, biomarker concentrations below the LoQ were replaced with the LoQ divided by the square root of 2. For comparison of serum cardiac biomarkers among groups, Mann–Whitney U test and Kruskal–Wallis followed by Dunn post-hoc test were used. Categorical variables were compared with the χ^2^ test. Spearman’s correlation was used to assess correlations between continuous variables. Two-way ANCOVA was used to explore sex-related differences in the serum levels of the cardiac biomarkers among groups. Multivariate ANCOVAs using log-transformed concentrations of sST2, NT-proBNP, and hs-cTnI as dependent variables, T2DM complications as factors, and age, sex, and HbA1c as covariates were constructed to identify factors associated with T2DM complications and treatments. Univariate tests for post-hoc comparisons are reported. Arrows indicate significant increase of the dependent variable with complications/treatments. The association between cardiac biomarker (sST2, NT-proBNP, and Dimension Vista hs-cTnI) levels and the follow-up endpoints was investigated by Kaplan-Meier curves and Cox proportional hazards analysis (adjusted for sex, age, smoking status, hypertension, T2DM duration, BMI, HbA1c, blood lipids, eGFR, and hs-CRP) with 95% confidence intervals. Logistic regressions were used for evaluating the associations with the endpoints encompassing MACE, as most of the events were not datable with precision. Optimal sST2, hs-cTnI, and NT-proBNP cut-offs for predicting survival in T2DM patients were computed using the Evaluate Cutpoints R package [24]. Biomarkers were fitted into the model as continuous or categorized variables. Based on the computed cutoffs, each biomarker was categorized into low, medium, and high. A score of 1–3 was assigned to the level of each variable (1 = low, 2 = medium, 3 = high, according to the previously defined cutoffs) and points assigned to each of the three biomarkers were summed to obtain a ‘cardiac score’, which ranged from 3 to 9. A sensitivity analysis was performed by assessing a subset of 238 T2DM samples using the alternative Abbott Architect i-STAT High-Sensitive Troponin I assay. Cox proportional hazard analysis was performed as aforementioned including only those samples tested with the Architect hs-cTnI method. Results of the two methods were compared using Bland–Altman plots and the Passing-Bablok regression. Only for method comparison, Architect hs-cTnI concentrations below the Dimension Vista hs-cTnI LoQ were substituted with the Dimension Vista LoQ divided by the square root of 2. Based on the results of the multivariable Cox regression analysis, a nomogram for predicting 5-, 10- and 15-year survival in type 2 diabetes was built using the “hdnom” package (version 6.0.0) for R [25]. Specifically, a penalized Cox regression model trained with an adaptive LASSO procedure using tenfold cross-validation was performed on the whole sample. Nomogram performance was assessed on 100 bootstrap samples and on a validation set including one third of the original samples. The predictive efficacy was assessed with internal and external calibration and discrimination statistics. Reclassification was assessed by using the continuous net reclassification improvement (NRI^>0^) [26]. Significance was accepted as p < 0.05. All data were analyzed using R (version 4.1), the Jamovi software (version 2.3.1), and the SPSS 26.0 for Windows software (SPSS Inc.; Chicago, IL, USA).

## Results

### Baseline subject’s characteristics and outcomes

Serum samples from a total of 683 subjects, including 568 patients with type 2 diabetes (T2DM) and 115 age- and sex-matched presumably healthy controls (CTR) were analyzed. Baseline subject’s characteristics are reported in Table 1. The two groups of subjects were significantly different for a number of variables related to anthropometrical data, lipid profile, and blood glucose control. After a median follow-up of 16.8 (IQR, 13.1–16.8) years, 202 patients had died (35.6%) and 7 patients (1.2%) were lost to follow-up. The mean survival time was 14.3 (95% CI 13.9–14.7) years. At the time of enrolment, 309 (54.4%) patients had at least one complication. Among 259 patients (45.6%) with uncomplicated T2DM at the time of enrolment, 159 patients (61.4%) developed at least one complication. Survival was higher in T2DM patients without complications compared to patients with at least one complication (log-rank test, p < 0.001; Additional file 1: Figure S1).

**Table 1:** Comparison of biochemical and anthropometric characteristics between healthy control subjects (CTR) and patients with type 2 diabetes mellitus (T2DM)

Variables	CTR N = 115	T2DM N = 568	p-value
Age (years)	68 (62–73)	67 (61–72)	0.215
Sex (Males %)	62 (54%)	308 (54%)	0.951
Current smoking (n %)	19 (17%)	84 (15%)	0.641
BMI (Kg/m^2^)	26.5 (23.9–29.0)	28.1 (25.8–31.4)	< 0.001
Weight (Kg)	73 (64–81)	77 (69.0–86.0)	< 0.001
Waist-hip ratio	0.91 (0.86–0.96)	0.94 (0.89–0.98)	0.004
Total cholesterol (mg/dL)	218 (188–244)	206 (181–233)	0.006
HDL-C (mg/dL)	56 (46–67)	50 (42–60)	0.001
LDL-C (mg/dL)	128 (108–151)	114 (96–135)	< 0.001
Triglycerides (mg/dL)	93 (71–132)	116 (83–159)	< 0.001
ApoA1 (mg/dL)	172 (153–195)	163 (146–186)	0.024
ApoB (mg/dL)	101 (87–121)	100 (84–118)	0.399
Fasting glucose (mg/dL)	92 (88–99)	153 (133–183)	< 0.001
HbA1C (%)	5.7 (5.5–6.0)	7.3 (6.6–8.0)	< 0.001
Insulin (µUI/mL)	5.48 (3.67–7.32)	5.75 (3.69–8.72)	0.233
HOMA index	1.19 (0.80–1.69)	2.15 (1.39–3.59)	< 0.001
Hemoglobin (g/dL)	14.1 (13.4–14.9)	14.3 (13.4–15.2)	0.127
WBC (n/mm^3^)	6.1 (5.2–7.0)	6.6 (5.6–7.6)	0.004
Platelets (n/mm^3^)	227 (181–272)	210 (179–252)	0.047
hs-CRP (mg/L)	1.8 (0.9–3.2)	2.5 (1.2–4.7)	0.002
Fibrinogen (mg/dL)	286 (242–334)	298 (255–346)	0.203
Iron (µg/dL)	74.5 (62.3–100.0)	81.0 (64.8–96.3)	0.358
Ferritin (ng/mL)	98 (53–152)	89 (47–162)	0.892
Creatinine (mg/dL)	0.8 (0.7–1.0)	0.9 (0.7–1.0)	0.050
eGFR (mL/min)	82 (70–98)	81 (66–87)	0.095
Azotemia (mg/dL)	39 (32–48)	38 (32–46)	0.564
Uric acid (mg/dL)	4.9 (4.1–5.7)	4.6 (4.0–5.5)	0.142
Alanine aminotransferase (U/L)	37 (33–42)	39 (34–48)	0.021
Aspartate aminotransferase (U/L)	22 (19–26)	20 (16–24)	0.001
Total bilirubin (mg/dL)	0.7 (0.6–0.9)	0.6 (0.5–0.8)	0.004
Gamma-glutamiltransferase (U/L)	49 (42–60)	50 (39–62)	0.800
Disease duration (years)	–	13 (7–22)	–
Relevant medications (n)			
Any T2DM medication	–	426 (75%)	–
Metformin	–	207 (36%)	–
Sulphonylureas	–	273 (48%)	–
Glinides	–	13 (2%)	–
Insulin	–	101 (18%)	–
Statins	–	109 (19%)	–
Vitamin K antagonists	–	46 (8%)	–
T2DM complications (n)			
Retinopathy	–	156 (27%)	–
Nephropathy	–	74 (13%)	–
Neuropathy	–	103 (18%)	–
History of MACE	–	84 (15%)	–
Peripheral artery disease	–	53 (9%)	–

Data are median (IQR) or number (%). P-values for Mann–Whitney U test

### Analysis of baseline sST2, hs-cTnI, and NT-proBNP in healthy controls and patients with type 2 diabetes

**Fig. 1:**
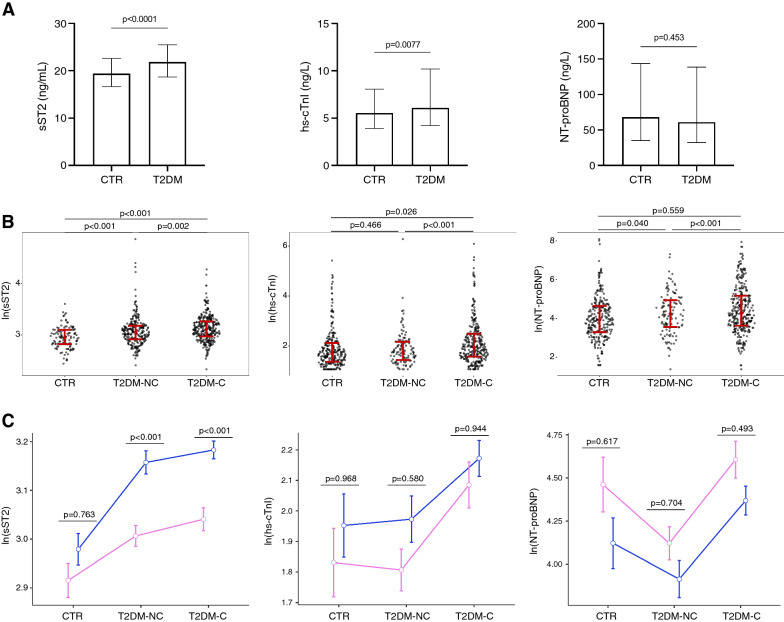
**A** Comparison of serum sST2, Dimension Vista hs-cTnI, and NT-proBNP between healthy controls (CTR) and patients with type 2 diabetes (T2DM). Data are median and IQR. P-values for Mann–Whitney *U* test. **B** Distribution of log-transformed sST2, hs-cTnI, and NT-proBNP among CTR and patients with uncomplicated (T2DM-NC) or complicated (T2DM-C) diabetes. P-values for Dunn’s post-hoc tests. **C** Log-transformed sST2, hs-cTnI, and NT-proBNP in CTR, T2DM-NC, and T2DM-C grouped according to sex. P-values for post-hoc Tukey test following two-way ANOVA.

Median serum sST2 (21.8 vs 19.1 ng/mL, p < 0.0001) and hs-cTnI (6.1 vs 5.7 ng/L, p = 0.0077) levels were higher in T2DM patients compared to CTR, while no significant difference was observed for NT-proBNP (62 vs. 69 ng/L, p = 0.453) (Fig. 1A). As expected, the distributions of the concentrations were highly right-skewed (coefficient of skewness, sST2 = 5.33, hs-cTnI = 9.38, NT-proBNP = 5.64; Additional file 1: Figure S2). Therefore, for the subsequent analyses, the three variables were log-transformed. Raw and transformed concentrations are reported in Additional file 1: Table S1.

When T2DM patients were categorized according to the presence of complications, ln(sST2) followed an increasing trend, with the highest values in the group of T2DM with complications (T2DM-C) (p < 0.001). A significant increase in ln(cTnI) values was observed in T2DM-C patients compared to T2DM patients without complications (T2DM-NC) (p < 0.001) and CTR (p = 0.026), whereas no significant difference was observed between T2DM-NC and CTR (p = 0.466). Regarding NT-proBNP, its levels were higher in CTR compared to T2DM-NC (p = 0.040) and in T2DM-C compared to T2DM-NC (p < 0.001). The levels of each biomarker across groups are shown in Fig. 1B. When exploring for sex-specific differences, we observed that ln(sST2) was higher in males with T2DM (two-way ANOVA, p < 0.001). On the other hand, ln (NT-proBNP) was significantly higher in females (p = 0.008), and no significant sex-related differences were observed for ln (cTnI) (p = 0.071) (Fig. 1C).

**Fig. 2:**
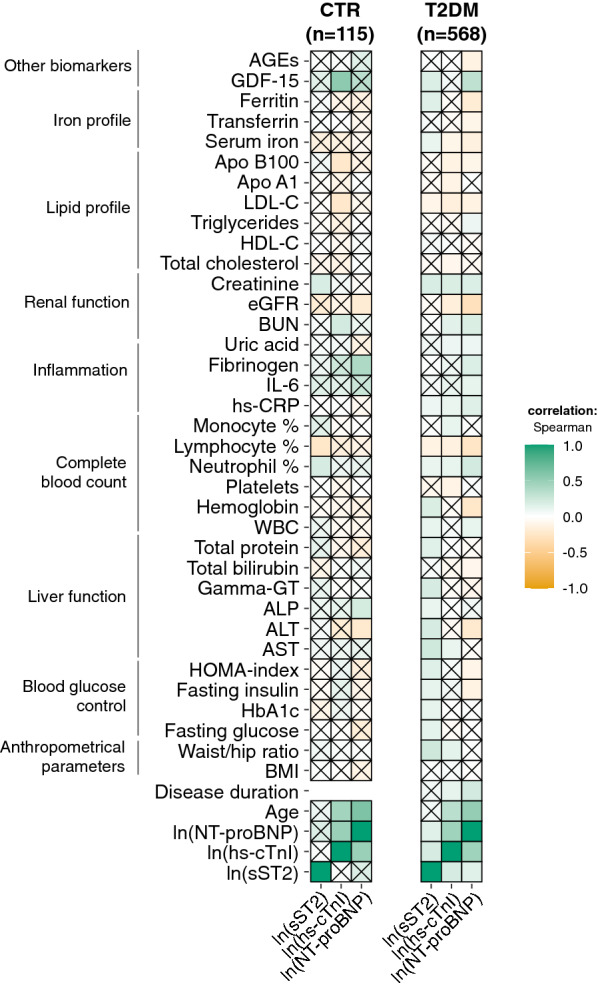
Spearman’s correlation plots for CTR subjects and patients with T2DM. The intensity of the color depends on the magnitude of the correlation. Non-significant correlations are crossed.

We then explored the correlations between the three cardiac biomarkers, expressed as ln, and the available biochemical variables. As expected, the three biomarkers showed a significant degree of reciprocal correlation, with hs-cTnI and NT-proBNP having the highest correlation coefficient (Spearman’s rho = 0.47, p < 0.001). The correlation plots in Fig. 2 summarize Spearman’s correlation coefficients for each pair of variables in the CTR and T2DM groups. The complete correlation matrix is available as Additional file 1: Table S2. Notably, hs-cTnI and NT-proBNP, but not sST2, showed a significant positive correlation with age. These correlations with age were particularly evident in CTR subjects (hs-cTnI, ρ = 0.29, p = 0.001; NT-proBNP, ρ = 0.59, p < 0.001), whereas weaker correlations were observed in T2DM patients (hs-cTnI, ρ = 0.26, p < 0.001; NT-proBNP, ρ = 0.49, p < 0.001). Only hs-cTnI and NT-proBNP showed a significant positive correlation with disease duration. Notably, sST2 was the only biomarker to show a significant pattern of positive correlations with variables related to blood glucose control, including fasting glucose, HbA1c, and HOMA-index, and liver function tests in T2DM. Moreover, positive correlations were observed for sST2 and hs-cTnI with the waist-hip ratio in T2DM. hs-cTnI and NT-proBNP levels increased with worsening renal function, while hs-cTnI andto a lesser extent NT-proBNP, showed a clear pattern of association with lipid profile, with inverse correlations with LDL-C and total cholesterol. Regarding the systemic inflammatory status, the three biomarkers were directly related to hs-CRP and neutrophil %, and inversely related to lymphocyte %. NT-proBNP was also directly related to IL-6 and fibrinogen. Notably, sST2 and NT-proBNP were positively related to the plasma concentration of GDF-15, an innovative biomarker of heart failure [27] which we previously assessed in a subgroup of patients [28].

To explore the effect of each T2DM complication and treatment on the levels of sST2, hs-cTnI, and NT-proBNP, multiple MANCOVA tests followed by one-way ANOVAs with Tukey’s post-hoc tests were computed after adjustment for age, sex, and HbA1c (Additional file 1: Table S3). NT-proBNP levels were higher in patients with either complication, hs-cTnI was higher in all T2DM complications except for nephropathy. Notably, sST2 levels, which were higher in complicated T2DM, were not affected by the presence of specific complications. Regarding T2DM treatments, higher levels of the three biomarkers were observed in patients under insulin therapy.

### Prognostic value of sST2, hs-cTnI, and NT-proBNP in type 2 diabetes

Subsequently, we aimed at evaluating whether sST2, hs-cTnI, and NT-proBNP, alone or in combination, were able to predict 15-year survival in T2DM patients and whether they added significantly to a reference model including the most relevant clinical and biochemical predictors, i.e. sex, age, smoking status, hypertension, T2DM duration, BMI, HbA1c, blood lipids, eGFR, and hs-CRP, using Kaplan-Maier and univariable and multivariable Cox regression methods. First, sST2, hs-cTnI, and NT-proBNP were categorized into 3 groups (i.e., low, medium, and high). The cutoffs for T2DM patients, computed to maximize the differences in survival prediction between groups, were as follows: sST2; low, < 16.5 ng/mL; high, ≥ 32.0 ng/mL; hs-cTnI; low, < 4.2 ng/L; high, ≥ 7.8 ng/L; NT-proBNP; low, < 89 ng/L; high, ≥ 337 ng/L.

**Fig. 3:**
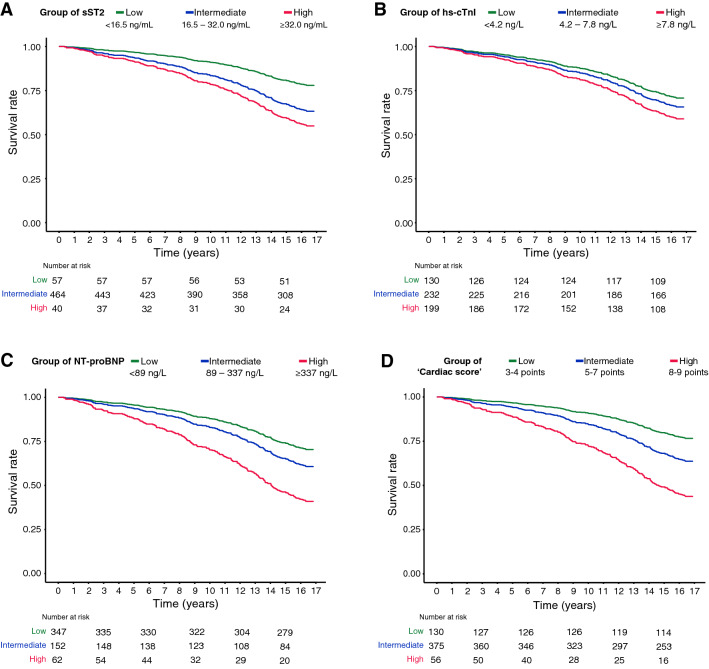
Kaplan–Meier survival estimates for **A** sST2, **B** Dimension Vista hs-cTnI, **C** NT-proBNP, **D** ‘Cardiac score’. Models adjusted for sex, age, smoking status, hypertension, T2DM duration, BMI, HbA1c, blood lipids, eGFR, and hs-CRP

All the cardiac biomarkers showed significant correlations with all-cause mortality in T2DM at the univariate analysis (Table 2). Multiple Cox regressions, adjusted for the confounders, revealed a significant association between sST2 and mortality in T2DM patients (model C-statistic = 0.729, 95% CI 0.696–0.762). Specifically, patients with high sST2 levels had an about threefold increased risk of death compared to patients with low serum levels. Similarly, an increased risk was observed in patients with high levels of hs-cTnI (C-statistic = 0.729, 95% CI 0.694 –0.764). Regarding NT-proBNP (C-statistic = 0.741, 95% CI 0.706–0.776) individuals with intermediate and higher levels had about 1.5- and 3-fold increased risk of death, respectively. Corresponding Kaplan–Meier curves are shown in Fig. 3A, B, C

**Table 2:** Summary of multiple Cox regression analysis of each biomarker and the combined ‘cardiac score’ for the prediction of survival in T2DM patients

Variable		N	Crude	Adjusted	Multimarker
			HR (95% CI)	HR (95% CI)	HR (95% CI)
Group of sST2	Low	57	Ref.	Ref.	Ref.
	Intermediate	464	**2.82 (1.45–5.52)**	**2.00 (1.02–3.94)**	**2.06 (1.04–4.06)**
	High	40	**3.77 (1.69–8.38)**	**2.76 (1.20–6.33)**	**2.34 (1.02–5.40)**
Group of hs-cTnI	Low	130	Ref.	Ref.	Ref.
	Intermediate	232	**1.73 (1.10–2.70)**	1.25 (0.78–1.99)	1.27 (0.79–2.03)
	High	199	**3.20 (2.08–4.93)**	**1.63 (1.01–2.62)**	1.33 (0.81–2.18)
Group of NT-proBNP	Low	347	Ref.	Ref.	Ref.
	Intermediate	152	**2.36 (1.72–3.23)**	**1.48 (1.05–2.10)**	1.40 (0.98–1.99)
	High	62	**5.47 (3.78–7.90)**	**2.94 (1.90–4.55)**	**2.76 (1.74–4.40)**
Group of ‘cardiac score’	3–4	130	Ref.	–	Ref.
	5–7	375	**2.74 (1.72–4.39)**	–	**1.79 (1.09–2.91)**
	8–9	56	**9.58 (5.60–16.40)**	–	**3.61 (1.95–6.68)**
‘Cardiac score’			**1.66 (1.49–1.85)**	–	**1.35 (1.19–1.53)**

Crude, adjusted (for sex, age, smoking status, hypertension, T2DM duration, BMI, HbA1c, blood lipids, eGFR, and hs-CRP), and multimarker hazard ratios (HR) with 95% confidence intervals are shown. Significant predictors are in bold. For the calculation of the ‘cardiac score’ the reader is referred to the Materials and Methods section

To enhance the predictive accuracy of the three biomarkers, we assigned a score to the level of each marker (1 = low, 2 = medium, 3 = high, according to the previously defined cutoffs) and computed an overall measure, ranging from 3 to 9, by summing the scores assigned to each variable. The combined ‘cardiac score’ was independently associated with the all-cause mortality risk (C-statistic = 0.739, 95% CI: 0.706–0.772), with patients in the ‘intermediate’ category having an about twofold increased risk of death and patients in the ‘high’ category having an about 3.5-fold increased risk of death (Table 2). The corresponding Kaplan–Meier curves are displayed in Fig. 3D. Moreover, when considering the ‘cardiac score’ as a continuous variable, each point-increase was associated with a significantly increased risk of death (HR: 1.35, 95%CI 1.19–1.53). All the three biomarkers, as well as the cardiac score, improved the C-statistic of the reference model (Additional file 1: Table S4). The category-free, continuous NRIs^>0^ of the predictive model obtained by adding the cardiac biomarkers as categorical variables to the classical risk factor model were 0.402 (95%CI 0.093–0.717), 0.302 (95% CI 0.105–0.493), and 0.335 (95%CI 0.081–0.586) at 5, 10, and 15 years of follow-up, respectively.

**Fig. 4:**
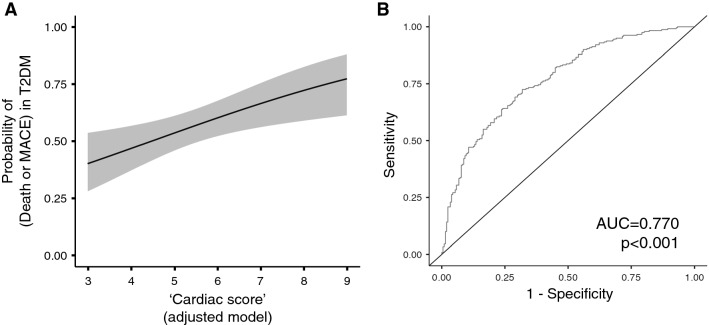
**A** Marginal means plot showing the probability of developing the composite endpoint death or MACE in T2DM patients based on the logistic regression model including ‘cardiac score’ as predictor (95% CI in gray). **B** ROC curve for the logistic regression model.

The combined score was tested also with the composite endpoint of all-cause death or MACE in T2DM patients without prior history of MACE. A binomial logistic regression, adjusted for sex, age, BMI, HbA1c, blood lipids, eGFR, and hs-CRP, revealed that the score is associated with increased odds of all-cause mortality or MACE (for 1-point increase, OR: 1.34, 95%CI 1.11–1.62) (Additional file 1: Table S5). The plot in Fig. 4A shows the probability of developing the outcome according to the score, while the ROC curve for the model (sensitivity, 75.8%; specificity, 62.8%; AUC: 0.770) is displayed in Fig. 4B. None of the biomarkers, nor the combination of them, was associated with an increased risk of developing each T2DM complication (data not shown).

### Comparison of troponin I assays

As an additional sensitivity analysis, a subset of 238 T2DM samples was tested with the Abbott Architect i-STAT High-Sensitive Troponin I assay, which received the CE clearance for the prediction of MACE in individuals without clinical signs of heart disease. The distribution of the hs-cTnI concentrations is displayed in Additional file 1: Figure S3A. The hs-cTnI concentrations ranged from 1.1 to 67.6 ng/L. Mean hs-cTnI concentration was 8.8 ng/L, median concentration was 5.8 ng/L (IQR 3.9–9.2 ng/L). A comparison of the two hs-cTnI assays using a Passing-Bablok regression revealed a good degree of correlation (y = 0.13 + 0.94 x, 95% CI slope 0.83–1.07, 95% CI intercept − 0.47–0.55); Additional file 1: Figure S3B). Bland–Altman analysis revealed a mean bias of 3.7 ng/L (95%CI 1.3–6.2 ng/mL) in favor of the Dimension Vista assay. The percentage bias was 7.7%. The lower limit of agreement (LLA) was -34.0 ng/L and the upper limit of agreement (ULA) was 41.4 ng/L (Additional file 1: Figure S3C). After filtering out samples with a mean of the two assays > 40 ng/L (resulting n = 224), a not significant bias of 0.2 ng/L (95% CI -0.8–1.1) was observed. The percentage bias was 2.4%, and the LLA and ULA were -13.7 ng/L and 14.0 ng/L, respectively (Additional file 1: Figure S3D).

The ability of the Architect hs-cTnI assay to predict survival in T2DM patients was also tested. First, two cut points, i.e. 4.4 ng/L and 7.5 ng/L were computed based on the best predictive performance. Therefore, hs-cTnI, recoded as a categorical variable (low, medium, high), was fit into the adjusted Cox regression model, which was computed only on those samples which were assessed with the Architect method. Similarly to the Dimension Vista hs-cTnI assay, an increased risk of death was observed in patients with high hs-cTnI-AB levels (≥ 7.5 ng/L) compared to patients with low levels (< 4.4 ng/L) (HR: 2.97, 95%CI 1.52–5.79), while no increase in mortality risk was observed with patients with intermediate levels **(**Additional file 1: Table S6**)**. Interestingly, also when T2DM patients were stratified according to the sex-specific risk categories provided by the manufacturer, a significantly increased risk of mortality was confirmed for patients in the high-risk category (HR: 2.10, 95%CI 1.11–4.00).

### Predictive nomogram

**Fig. 5:**
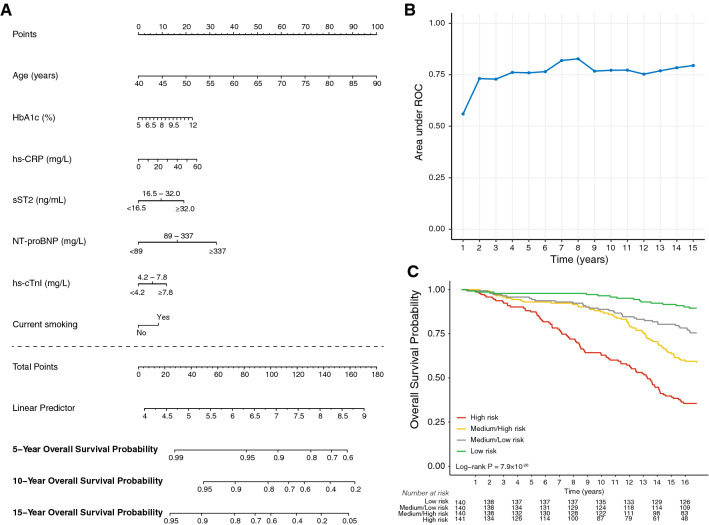
**A** Nomogram for predicting overall survival in patients with type 2 diabetes. The points assigned to each variable are summed up to obtain the total score and a vertical line can be drawn to obtain the corresponding survival probability. **B** Performance of the model based on the external validation dataset. Model areas under the curve (AUCs) at each year are displayed. **C** Kaplan–Meier survival function for patients with type 2 diabetes according to quartiles of the nomogram-based mortality risk score

Based on the results of the Cox regression models, an adaptive LASSO regression model and multivariate Cox regression analysis were computed to predict survival at 5, 10, and 15 years in type 2 diabetes based on the following variables: age, sex, HbA1c, eGFR, blood lipids, hs-CRP, and the cardiac biomarkers sST2, Dimension Vista hs-cTnI, and NT-proBNP as categorical variables. For this purpose, the sample was randomly split into training (n = 375) and validation (n = 186) datasets, with approximately equal proportions of deceased individuals. Sex, eGFR, and blood lipids were excluded by the LASSO procedure. Figure 5A presents the model in the form of a nomogram that provides the long-term survival probabilities corresponding to a particular total score. The total score for a patient is obtained by adding up the scores for each of the seven predictors. Internal validation with bootstrap resampling performed on the training dataset showed that the AUCs for the all-cause mortality prediction nomogram were 0.685, 0.770 and 0.758 at 5, 10 and 15 years respectively (Additional file 1: Figure S4A), whereas the AUCs for the validation dataset were 0.760, 0.772 and 0.795 at 5, 10 and 15 years respectively (Fig. 5B). The internal and external calibration plots revealed a good agreement and correlation between the observed and predicted values (Additional file 1: Figure S4B). Based on the risk profile predicted by the nomogram, 4 homogeneous groups of patients were generated, and Kaplan–Meier survival curves were constructed (Fig. 5C). The log-rank test confirmed that the survival curves of patients grouped according to the nomogram-based mortality risk score were significantly different (p = 7.9 × 10^–26^).

## Discussion

In our retrospective study, we observed that sST2 and, to a lesser extent NT-proBNP, followed an increasing trend from CTR to T2DM patients with at least one complication (T2DM-C). On the other hand, an increase in hs-cTnI levels, assessed using the Dimension Vista method, was evident only in T2DM-C. Previous observational studies showed the association of sST2 with T2DM, although conflicting results were reported regarding disease severity in terms of complications. Fousteris et al. showed that sST2 was higher in T2DM patients compared to healthy controls, however, no difference was observed for T2DM patients grouped according to the presence of left ventricular diastolic dysfunction [29]. These results were confirmed by a broader evaluation of serum sST2 in 3′450 participants of the Framingham Heart Study Offspring cohort [30]. Notably, sST2 was the only biomarker showing a clear association with blood glucose control in T2DM, in particular a direct correlation with fasting glucose, HbA1c, and HOMA-index, in line with previous reports on smaller cohorts of T2DM [20] and healthy [31] individuals. Moreover, an exclusive pattern of positive correlations between sST2 and markers of liver function, such as AST, ALT, gamma-GT, and ALP was observed in T2DM patients. In liver disease, as observed during liver fibrosis and hepatitis B [32], stressed hepatocytes release IL-33, which was demonstrated to protect hepatocytes by simultaneously repressing the expression of proapoptotic genes and activating antiapoptotic genes [33]. Therefore, it is conceivable that in patients with T2DM the high levels of sST2 could attenuate this pathway, thus accelerating the accumulation of liver damage related to insulin resistance.

Regarding renal function, we observed increased levels of hs-cTnI and NT-proBNP with declining eGFR, and higher levels of the latter in diabetic nephropathy, in agreement with several previous reports [34–36]. While NT-proBNP accumulation with worsening renal function poses an intrinsic limitation of this biomarker, it was shown that the decline in kidney function during therapy for acute decompensated heart failure is associated with improved outcomes as long as NT-proBNP levels are decreasing, supporting the incorporation of congestive HF biomarker as guidance for interpreting acute declines in kidney function among patients admitted for acute decompensated HF [37]. On the opposite, sST2 was not associated with renal function nor with the presence of diabetic nephropathy. This peculiar characteristic paved the way for studies assessing the prognostic relevance of sST2 in patients with renal insufficiency. Indeed, sST2 was proven to be an independent prognostic marker [38, 39], and a predictor of incident HF in patients with chronic kidney disease [40].

When exploring specific T2DM complications, NT-proBNP and hs-cTnI were associated with neuropathy, atherosclerotic vascular disease, and retinopathy, independent of age, sex, and blood glucose control. Interestingly, the association between hs-cTnI and diabetic neuropathy was investigated by a recent study showing that hs-TnT is negatively related to sciatic nerve integrity and positively correlated with the neuropathy disability score [41]. NT-proBNP and hs-cTnI were higher in patients with a history of MACE, confirming the integrity of samples after storage and the validity of the anamnestic information.

We performed a longitudinal evaluation of T2DM patients to assess whether the cardiac biomarkers sST2, Dimension Vista hs-cTnI, and NT-proBNP were associated with 15-year all-cause mortality and development of MACE and fitted the overall model into a nomogram to predict 5-, 10-, and 15-year survival. Notably, all three biomarkers independently predicted mortality after adjustment for the most relevant CV risk factors. Specifically, patients with serum sST2 ≥ 32 ng/mL had a 2.8-fold increased risk of death compared to patients with sST2 < 16.5 ng/mL. The two cutoff values were computed to maximize the significance of the prediction. Notably, our cutoff to identify individuals at the highest risk was similar to the 35 ng/mL cutoff identified for the prediction of poor outcomes in chronic HF [42]. While several reports are available on the prognostic value of sST2 in patients with established CVD, to our knowledge this is the first study evaluating the long-term prognostic value of sST2 in T2DM patients. Data from the Tor Vergata Atherosclerosis Registry showed that high sST2 levels predict all-cause and CV mortality in a cohort of 399 patients with atherosclerotic disease (median follow-up of 75 months), including subjects with impaired glucose tolerance of T2DM, which had the highest sST2 levels [20]. A short-term (180 days) follow-up study on patients with T2DM and HD showed that sST2 levels > 54 ng/mL were associated with a higher risk of death and rehospitalization for cardiovascular causes [43]. Another study showed that plasma sST2 levels ≥ 19 ng/mL significantly predicted the development of MACEs and other CV endpoints in patients with CAD after adjusting for age, sex, and presence of T2DM [19].

As expected, our data confirmed that also NT-proBNP and hs-cTnI were independently associated with all-cause mortality in T2DM, albeit the association with hs-cTnI was lost in the model encompassing all three biomarkers. NT‐proBNP was shown to be as discriminatory as a model of 20 traditional clinical and laboratory variables in the prediction of both death and cardiovascular events in high-risk patients with T2DM [44]. A secondary analysis of the ADVANCE trial showed that NT-proBNP and hs-cTnT were strongly associated with an increased risk of MACE and death over a 5 year follow-up period, even after extensive adjustment for established and emerging clinical predictors of outcome in T2DM [45]. Finally, following the observation that each of these biomarkers was related to specific biochemical features of the disease, we computed a combined ‘cardiac score’ by summing up the points attributed to the level of each biomarker (low, medium, or high). Notably, the obtained score was a significant independent predictor of mortality in T2DM. Patients with a score ≥ 8, which corresponds to at least two biomarkers in the highest values, had a 3.6-fold increased risk of death, while the risk was increased by 1.8-fold in patients with a score between 5 and 7. The score was also independently associated with the composite endpoint determined by all-cause mortality and MACE in T2DM patients without previous history of MACE. While this combined approach has been extensively reported for the prognostic stratification of patients with established CVD [46–48], no data is available so far in clinically heterogeneous cohorts of T2DM patients.

Finally, the comparison of two different hs-cTnI methods, i.e., the Dimension Vista^®^ and the Architect i-Stat assays, revealed a good concordance, with a negligible bias for mean hs-cTnI concentrations below 40 ng/mL, as observed in > 90% of the tested samples. It must be noted, however, that significant differences were previously reported between the two hs-cTnI methods used in this study, concerning the analytical design of immunometric systems, the analytical performance, and the measured biomarker concentration, in particular in terms of 99th percentile values [49]. These differences in the analytical characteristics and clinical performance between the Architect and Siemens immunoassays prevent a good harmonization of the measured biomarker concentrations, unless specific recalibration procedures are applied [50]. For these reasons, hs-cTnI concentrations obtained with different methods were analyzed separately in this study. The Architect assay, when considered as a categorical variable, was associated with mortality in T2DM, with individuals in the group with the highest levels showing a 3-fold increased risk of death. Our study confirms the results of the assessment of hs-cTnI using the Architect assay in a subset of 1'704 T2DM patients from the Atherosclerosis Risk in Communities (ARIC) study, which showed that hs-cTnI was independently associated with and significantly improved model discrimination for all-cause and cardiovascular mortality risk [51].

The present study has limitations that need to be addressed. First, none of the patients was treated with the novel antidiabetic drugs at the time of enrolment, and their progressive adoption over the follow-up period could have impacted significantly on the outcomes. Second, the glycemic and lipid targets were enforced over time, thus affecting the burden of CV risk factors in our patients. Third, no intermediate information was available on the degree of glycemic control and on biochemical variables related to T2DM complications (e.g. urinary albumin-to-creatinine ratio), as well as on the mortality outcome for CTR subjects. However, the enrolled T2DM patients were constantly followed by a dedicated facility, with strong adherence to the latest standards of care and with periodic monitoring for the development of complications, as confirmed by the very small proportion of lost to follow-up patients (1.2%). Importantly, anamnestic information on the previous history of MACE and CV medications was available at the time of enrolment. Samples were collected and stored at − 80 °C for the entire follow-up period and dedicated serum aliquots were used only for the present study. Regarding the follow-up of CTR subjects, information on survival and causes of death will be collected in the near future, thus allowing us to evaluate how T2DM affects the ability of cardiac biomarkers to predict survival.

## Conclusion

Findings from the present study confirmed that sST2, hs-cTnI, and NT-proBNP are associated with mortality and the onset of MACE in patients with type 2 diabetes. Their ability to track different features of the disease, including insulin resistance and associated metabolic disorders, and their additive value in predicting survival support their routine assessment in the follow-up of T2DM patients. Moreover, the ability to partly overcome some of the limitations of the conventional cardiac biomarkers and the availability of a CE-IVD-marked fully automated assay promotes the adoption of sST2 as a biomarker of CV risk in healthy individuals and in the context of diseases associated with increased CV risk.

## Supplementary Information

**Additional file 1: Table S1. **Comparison of raw and log-transformed sST2, hs-cTnI, and NT-proBNP serum levels between healthy control subjects (CTR) and patients with type 2 diabetes mellitus (T2DM). Data are median (IQR). P-values for Mann-Whitney U test. **Table S2. **Correlation matrix between selected clinical/biochemical variables and serum sST2, hs-cTnI, and NT-proBNP in CTR (n=115) and T2DM (n=568) subjects. **Table S3. **Results of the MANCOVA model in which log-transformed concentrations of sST2, NT-proBNP, and hs-cTnI were used as dependent variables and each T2DM complication as factor. Age, sex, and HbA1c were used as covariates. Univariate tests for post-hoc comparisons are reported. Arrows indicate significant increase of the dependent variable with complications/treatments. **Table S4. **C-statistics, with 95% confidence intervals, of the Cox regression models for predicting all-cause mortality in T2DM patients. **Table S5. **Logistic regression model predicting likelihood of developing the composite endpoint death or MACE in T2DM patients without previous history of MACE. Model summary, χ²=107, df=12, p<0.001, Nagelkerke’s R2=0.284. **Table S6. **Summary of multiple Cox regression analysis for Architect hs-cTnI levels, categorized according to the best cutoffs (4.4 ng/L and 7.5 ng/L) and to the cardiovascular risk categories defined by the manufacturer, for the prediction of survival in T2DM patients. Crude, adjusted (for sex, age, smoking status, hypertension, T2DM duration, BMI, HbA1c, blood lipids, eGFR, and hs-CRP), and multimarker hazard ratios (HR) with 95% confidence intervals are shown. Significant predictors are in bold** Figure S1. **Kaplan-Meier survival estimates with 95% confidence intervals for patients with T2DM grouped according to the absence (No) or presence (Yes) of T2DM complications. **Figure S2. **Distribution of serum sST2, Dimension Vista hs-cTnI, and NT-proBNP serum among healthy controls (CTR) and patients with type 2 diabetes (T2DM). **Figure S3. A **Histogram showing the distribution of raw Architect hs-cTnI serum levels among patients with type 2 diabetes (T2DM). **B **Passing-Bablok regression between the Siemens Dimension Vista TNIH and the Abbott Architect i-STAT assays for the measurement of hs-cTnI. Regression line with 95% CI is displayed in blue. Line of identity is in red. **C**–**D **Bland-Altman plots showing differences between Dimension Vista TNIH and Abbott’s Architect i-STAT hs-cTnI assays for all samples **C**, and for mean hscTnI values <40 ng/L **D**. Lower and upper levels of agreement with 95% CI are displayed in red and green, respectively. **Figure S4. A **Performance of the model based on the internal validation. Model area under the curves (AUCs) at each year are displayed. The solid line represents the mean of the AUC, the dashed line represents the median of the AUC. The darker interval in the plot shows the 25% and 75% quantiles of AUC, the lighter interval shows the minimum and maximum of AUC. **B **Internal ed external calibration plots showing the observed against the predicted 15 year survival probability for patients with type 2 diabetes grouped according to risk quartiles. The grey diagonal line represents perfect calibration

## Data Availability

The datasets generated during and/or analyzed during the current study are available from the corresponding author on reasonable request.

## Abbreviations

T2DM: Type 2 diabetes mellitus
CV: Cardiovascular
BNP: Brain natriuretic peptide
cTn: Cardiac troponin
sST2: Soluble suppression of tumorigenesis 2
NT-proBNP: N-terminal proBNP
CTR: Control
UACR: Urinary-albumin-to-creatinine ratio
MACE: Major adverse cardiovascular events
HF: Heart failure
OGTT: Oral glucose tolerance test
eGFR: Estimated glomerular filtration rate
NRI: Net reclassification improvement
LASSO: Least absolute shrinkage and selection operator
