# Supplementary material for: Prognostic value of soluble ST2, high-sensitivity cardiac troponin, and NT-proBNP in type 2 diabetes: a 15-year retrospective study
Source: Cardiovasc Diabetol. 2022 Sep 10;21:180. doi: 10.1186/s12933-022-01616-3 (PMC9463761; doi:10.1186/s12933-022-01616-3)

SUPPLEMENTAL MATERIAL

Prognostic Value of Soluble ST2, High-Sensitivity Cardiac Troponin, and NT-proBNP in Type 2 Diabetes: a 15-Year Retrospective Study

SUPPLEMENTARY TABLES

Supplementary Table 1. Comparison of raw and log-transformed sST2, hs-cTnI, and NT-proBNP serum levels between healthy control subjects (CTR) and patients with type 2 diabetes mellitus (T2DM). Data are median (IQR). P-values for Mann-Whitney U test.

**Variables**	**CTR N=115**	**T2DM N=568**	**p-value**
sST2 (ng/ml)	19.1 (16.8 – 22.2)	21.8 (18.7 – 25.4)	<0.001
ln(sST2)	2.95 (2.82 – 3.10)	3.08 (2.93 – 3.24)	<0.001
hs-cTnI (ng/L)	5.7 (4.2 – 8.8)	6.1 (4.3 – 10.2)	0.689
ln(hs-cTnI)	1.74 (1.43 – 2.17)	1.81 (1.45 – 2.32)	0.138
NT-proBNP (ng/L)	69 (36 – 140)	62 (32 – 138)	0.305
ln(NT-proBNP)	4.23 (3.57 – 4.94)	4.11 (3.47 – 4.92)	0.896

Supplementary Table 2. Correlation matrix between selected clinical/biochemical variables and serum sST2, hs-cTnI, and NT-proBNP in CTR (n=115) and T2DM (n=568) subjects

		CTR			T2DM		
		ln(sST2)	ln(NT-proBNP)	ln (hs-cTnI)	ln(sST2)	ln(NT-proBNP)	ln (hs-cTnI)
ln(sST2)	Spearman's rho	—			—		
	p-value	—			—		
ln(NT-proBNP)	Spearman's rho	0.17	—		0.15	—	
	p-value	0.069	—		<.001	—	
ln(hs-cTnI)	Spearman's rho	0.026	0.492	—	0.196	0.463	—
	p-value	0.786	<.001	—	<.001	<.001	—
Age	Spearman's rho	0.087	0.624	0.451	0.063	0.524	0.332
	p-value	0.355	<.001	<.001	0.134	<.001	<.001
T2DM duration	Spearman's rho				0.075	0.223	0.118
	p-value				0.097	<.001	0.009
BMI	Spearman's rho	0.025	-0.106	-0.014	0.019	-0.015	0.035
	p-value	0.794	0.262	0.881	0.652	0.726	0.405
Waist/hip ratio	Spearman's rho	0.073	0.02	0.034	0.241	0.007	0.135
	p-value	0.439	0.829	0.72	<.001	0.874	0.001
Fasting glucose	Spearman's rho	0.008	-0.172	-0.011	0.133	0	-0.036
	p-value	0.933	0.066	0.911	0.001	0.999	0.398
HbA1c	Spearman's rho	-0.092	-0.033	0.082	0.121	0.017	0.002
	p-value	0.326	0.728	0.385	0.004	0.683	0.967
Fasting insulin	Spearman's rho	-0.036	-0.103	0.122	0.122	-0.115	0.071
	p-value	0.704	0.276	0.195	0.004	0.006	0.093
HOMA-index	Spearman's rho	-0.044	-0.152	0.092	0.165	-0.099	0.039
	p-value	0.639	0.104	0.327	<.001	0.018	0.348
AST	Spearman's rho	0.07	0.101	0.077	0.227	-0.017	0.097
	p-value	0.456	0.286	0.417	<.001	0.694	0.021
ALT	Spearman's rho	0.055	-0.208	-0.18	0.201	-0.208	-0.048
	p-value	0.564	0.027	0.055	<.001	<.001	0.252
Alkaline phosphatase	Spearman's rho	0.076	0.207	0.105	0.097	0.069	0.008
	p-value	0.421	0.027	0.263	0.02	0.102	0.85
Gamma-GT	Spearman's rho	0.101	0.016	-0.008	0.196	-0.08	-0.025
	p-value	0.285	0.87	0.935	<.001	0.058	0.552
Total bilirubin	Spearman's rho	-0.09	0.056	0.029	0.04	-0.083	-0.063
	p-value	0.341	0.555	0.76	0.341	0.048	0.136
Total proteins	Spearman's rho	0.122	-0.157	-0.078	0.147	-0.042	0.01
	p-value	0.197	0.095	0.411	<.001	0.321	0.819
White blood cells	Spearman's rho	0.084	-0.066	-0.05	0.106	0.112	0.038
	p-value	0.375	0.483	0.598	0.011	0.007	0.367
Hemoglobin	Spearman's rho	-0.037	-0.113	-0.073	0.181	-0.235	-0.027
	p-value	0.695	0.231	0.44	<.001	<.001	0.517
Platelets	Spearman's rho	0.013	-0.004	-0.069	-0.074	0.034	-0.111
	p-value	0.89	0.965	0.463	0.078	0.417	0.008
Neutrophil %	Spearman's rho	0.198	0.101	0.088	0.099	0.224	0.107
	p-value	0.035	0.285	0.351	0.018	<.001	0.011
Lymphocyte %	Spearman's rho	-0.244	-0.11	-0.149	-0.116	-0.252	-0.138
	p-value	0.009	0.243	0.115	0.006	<.001	<.001
Monocyte %	Spearman's rho	0.141	0.005	-0.037	0.019	0.02	0.108
	p-value	0.133	0.962	0.693	0.657	0.638	0.01
hs-CRP	Spearman's rho	-0.003	-0.064	0.021	0.092	0.161	0.102
	p-value	0.977	0.498	0.825	0.028	<.001	0.015
IL-6	Spearman's rho	0.146	0.256	0.141	0.047	0.119	0.1
	p-value	0.338	0.09	0.357	0.431	0.047	0.096
Fibrinogen	Spearman's rho	0.088	0.401	0.244	0.013	0.169	0.082
	p-value	0.488	0.001	0.052	0.77	<.001	0.057
Uric acid	Spearman's rho	0.044	-0.121	0.056	0.07	0.093	0.089
	p-value	0.641	0.199	0.55	0.094	0.026	0.035
Blood urea nitrogen	Spearman's rho	0.043	0.077	0.217	0.024	0.18	0.142
	p-value	0.646	0.41	0.02	0.575	<.001	<.001
eGFR	Spearman's rho	-0.182	-0.191	-0.099	-0.036	-0.292	-0.159
	p-value	0.052	0.041	0.292	0.393	<.001	<.001
Creatinine	Spearman's rho	0.192	-0.032	0.063	0.192	0.165	0.184
	p-value	0.04	0.732	0.501	<.001	<.001	<.001
Total cholesterol	Spearman's rho	-0.085	0.014	-0.11	-0.031	-0.052	-0.085
	p-value	0.366	0.882	0.244	0.46	0.22	0.043
HDL-C	Spearman's rho	-0.02	0.001	-0.119	-0.016	0.087	-0.054
	p-value	0.835	0.989	0.203	0.695	0.039	0.203
Triglycerides	Spearman's rho	-0.002	0.029	-0.056	0.042	-0.039	0.017
	p-value	0.982	0.76	0.553	0.317	0.358	0.68
LDL-C	Spearman's rho	-0.006	-0.072	-0.227	-0.099	-0.125	-0.151
	p-value	0.946	0.448	0.015	0.018	0.003	<.001
Apo A1	Spearman's rho	-0.018	0.012	-0.1	-0.037	0.011	-0.107
	p-value	0.849	0.896	0.29	0.378	0.803	0.011
Apo B	Spearman's rho	0.058	-0.111	-0.234	-0.037	-0.089	-0.123
	p-value	0.542	0.238	0.012	0.378	0.034	0.003
Serum iron	Spearman's rho	-0.142	-0.061	-0.129	0.099	-0.14	-0.085
	p-value	0.133	0.518	0.172	0.019	<.001	0.042
Transferrin	Spearman's rho	0.009	-0.082	-0.003	0.039	-0.089	-0.04
	p-value	0.921	0.387	0.978	0.359	0.034	0.338
Ferritin	Spearman's rho	0.036	-0.119	-0.102	0.16	-0.177	-0.03
	p-value	0.702	0.209	0.281	<.001	<.001	0.47
GDF-15	Spearman's rho	0.142	0.331	0.551	0.166	0.313	0.064
	p-value	0.431	0.06	<.001	0.008	<.001	0.309
AGEs	Spearman's rho	0.015	0.151	0.034	0.024	-0.122	-0.004
	p-value	0.91	0.262	0.803	0.567	0.004	0.926

Supplementary Table 3. Results of the MANCOVA model in which log-transformed concentrations of sST2, NT-proBNP, and hs-cTnI were used as dependent variables and each T2DM complication as factor. Age, sex, and HbA1c were used as covariates. Univariate tests for post-hoc comparisons are reported. Arrows indicate significant increase of the dependent variable with complications/treatments.

	Dependent Variable		Sum of Squares	df	Mean Square	F	p
T2DM complications							
**Peripheral artery disease** (Wilks' lambda=0.962, F[3,561]=7.30, p<0.001)	ln(sST2)		0.0503	1	0.0503	0.747	0.388
	ln(hs-cTnI)	↑	4.4638	1	4.4638	6.739	0.010
	ln(NT-proBNP)	↑	22.4410	1	22.4410	20.896	<0.001
**Major adverse cardiovascular events (MACE)** (Wilks' lambda=0.930, F[3,561]=14.06, p<0.001)	ln(sST2)		0.1335	1	0.1335	1.985	0.159
	ln(hs-cTnI)	↑	8.5207	1	8.5207	12.978	<0.001
	ln(NT-proBNP)	↑	41.5215	1	41.5215	39.954	<0.001
**Neuropathy** (Wilks' lambda=0.975, F[3,561]=4.84, p=0.002)	ln(sST2)		0.1256	1	0.1256	1.868	0.172
	ln(hs-cTnI)	↑	4.5545	1	4.5545	6.901	0.009
	ln(NT-proBNP)	↑	12.5419	1	12.5419	11.704	<0.001
**Nephropathy** (Wilks' lambda=0.959, F[3,561]=7.93, p<0.001)	ln(sST2)		0.1465	1	0.1465	2.179	0.140
	ln(hs-cTnI)		2.0010	1	2.0010	3.019	0.083
	ln(NT-proBNP)	↑	24.5990	1	24.5990	23.336	<0.001
**Retinopathy** (Wilks' lambda=0.977, F[3,561]=4.39, p=0.005)	ln(sST2)		0.0237	1	0.0237	0.3531	0.553
	ln(hs-cTnI)	↑	4.2379	1	4.2379	6.4240	0.012
	ln(NT-proBNP)	↑	11.9628	1	11.9628	11.110	<0.001
T2DM treatments							
**Metformin** (Wilks' lambda=0.998, F[3,561]=0.398, p=0.754)							
**Sulphonylureas** (Wilks' lambda=0.998, F[3,561]=0.427, p=0.734)							
**Glinides** (Wilks' lambda=0.991, F[3,561]=1.73, p=0.160)							
**Insulin** (Wilks' lambda=0.957, F[3,561]=8.47, p<0.001)	ln(sST2)	↑	0.2797	1	0.2797	4.1738	0.042
	ln(hs-cTnI)	↑	7.3519	1	7.3519	11.254	<0.001
	ln(NT-proBNP)	↑	21.5967	1	21.5967	20.253	<0.001

Supplementary Table 4. C-statistics, with 95% confidence intervals, of the Cox regression models for predicting all-cause mortality in T2DM patients.

**Model**	**C-statistic (95% CI)**
Reference model (sex, age, smoking status, hypertension, T2DM duration, BMI, HbA1c, blood lipids, eGFR, and hs-CRP)	0.723 (0.688 – 0.758)
Reference model + Group of sST2	0.729 (0.696 – 0.762)
Reference model + Group of Dimension Vista hs-cTnI	0.729 (0.694 – 0.764)
Reference model + Group of NT-proBNP	0.741 (0.706 – 0.776)
Reference model + 'Cardiac score'	0.739 (0.706 – 0.772)

Supplementary Table 5. Logistic regression model predicting likelihood of developing the composite endpoint death or MACE in T2DM patients without previous history of MACE. Model summary, χ^2=107, df=12, p<0.001, Nagelkerke's R^2=0.284.

						95% Confidence Interval	
**Predictor**	**Estimate**	**SE**	**Z**	**p**	**Odds ratio**	**Lower**	**Upper**
'Cardiac score'	0.29395	0.09711	3.0269	0.002	1.34171	1.109	1.623
Sex:							
M vs. F	0.33533	0.24271	1.3816	0.167	1.39840	0.869	2.250
Age	0.10018	0.01690	5.9274	< .001	1.10537	1.069	1.143
Current smoking	0.26328	0.30799	0.8548	0.393	1.30119	0.712	2.380
HbA1c	0.05713	0.09209	0.6203	0.535	1.05879	0.884	1.268
hs-CRP	0.03170	0.02412	1.3145	0.189	1.03221	0.985	1.082
Total cholesterol	0.00538	0.00639	0.8421	0.400	1.00539	0.993	1.018
LDL-C	-0.00721	0.00672	-1.0724	0.284	0.99282	0.980	1.006
HDL-C	-0.02820	0.01123	-2.5120	0.012	0.97219	0.951	0.994
Triglycerides	1.80e-4	0.00187	0.0963	0.923	1.00018	0.997	1.004
eGFR	-0.00778	0.00615	-1.2637	0.206	0.99225	0.980	1.004
BMI	-0.03449	0.02829	-1.2190	0.223	0.96610	0.914	1.021

Supplementary Table 6. Summary of multiple Cox regression analysis for Architect hs-cTnI levels, categorized according to the best cutoffs (4.4 ng/L and 7.5 ng/L) and to the cardiovascular risk categories defined by the manufacturer, for the prediction of survival in T2DM patients. Crude, adjusted (for sex, age, smoking status, hypertension, T2DM duration, BMI, HbA1c, blood lipids, eGFR, and hs-CRP), and multimarker hazard ratios (HR) with 95% confidence intervals are shown. Significant predictors are in bold.

			**Crude**	**Adjusted**	**Multimarker (+ sST2 and NT-proBNP)**
**Variable**		**N**	**HR (95% CI)**	**HR (95% CI)**	**HR (95% CI)**
**Levels of Architect hs-cTnI**	Low	76	Ref.	Ref.	Ref.
	Intermediate	76	1.60 (0.79 – 3.25)	1.16 (0.55 – 2.43)	1.15 (0.54 – 2.46)
	High	81	**4.78 (2.59 – 8.85)**	**2.97 (1.52 – 5.79)**	**2.80 (1.39 – 5.64)**
**Architect hs-cTnI risk category**	Low	95	Ref.	Ref.	Ref.
	Intermediate	98	**2.01 (1.16 – 3.47)**	1.29 (0.72 – 2.33)	1.29 (0.71 – 2.35)
	High	40	**3.76 (2.08 – 6.82)**	**2.10 (1.11 – 4.00)**	1.74 (0.88 – 3.45)
**Ln(Architect hs-cTnI)**			**1.99 (1.46 – 2.67)**	**1.56 (1.09 – 2.23)**	1.36 (0.94 – 1.96)

SUPPLEMENTARY FIGURES

Supplementary Figure 1. Kaplan-Meier survival estimates with 95% confidence intervals for patients with T2DM grouped according to the absence (No) or presence (Yes) of T2DM complications.

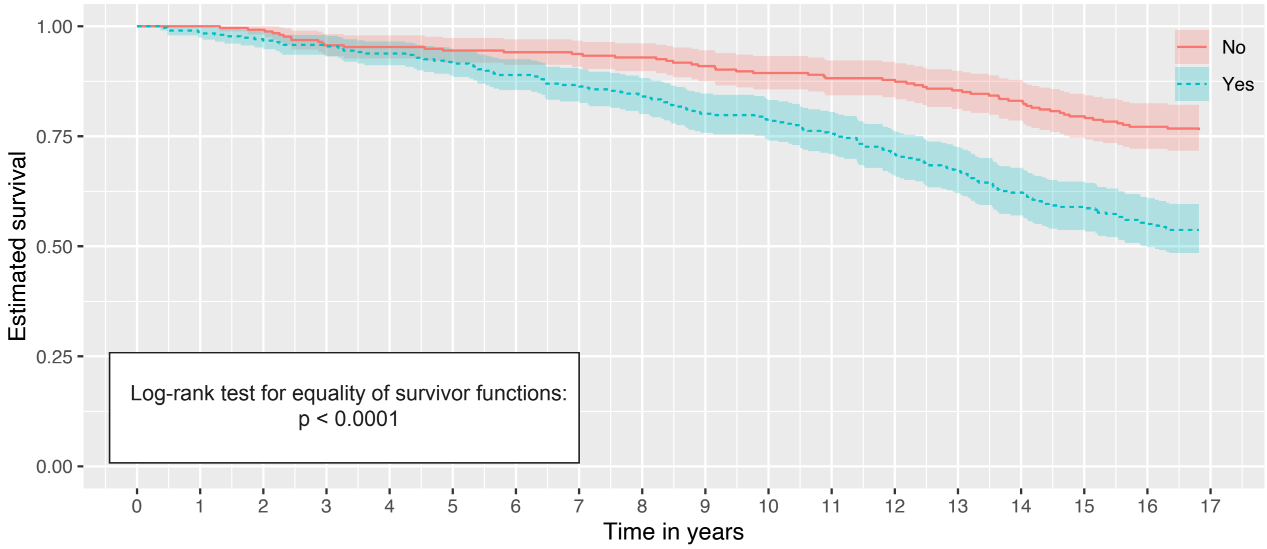

Absence of T2DM complications

At risk	254	254	252	243	242	240	239	238	236	231	227	224	223	217	211	202	196	0
Censored	0	0	0	0	0	0	0	0	0	0	0	0	0	0	0	0	0	194
Events	0	0	2	11	12	14	15	16	18	23	27	30	31	37	43	52	58	60

Presence of T2DM complications

At risk	307	303	297	294	288	281	273	266	258	246	241	232	218	206	191	181	169	0
Censored	0	0	0	0	0	0	0	0	0	0	0	0	0	0	0	0	0	165
Events	0	4	10	13	19	26	34	41	49	61	66	75	89	101	116	126	138	142

Supplementary Figure 2. Distribution of serum sST2, Dimension Vista hs-cTnI, and NT-proBNP serum among healthy controls (CTR) and patients with type 2 diabetes (T2DM).

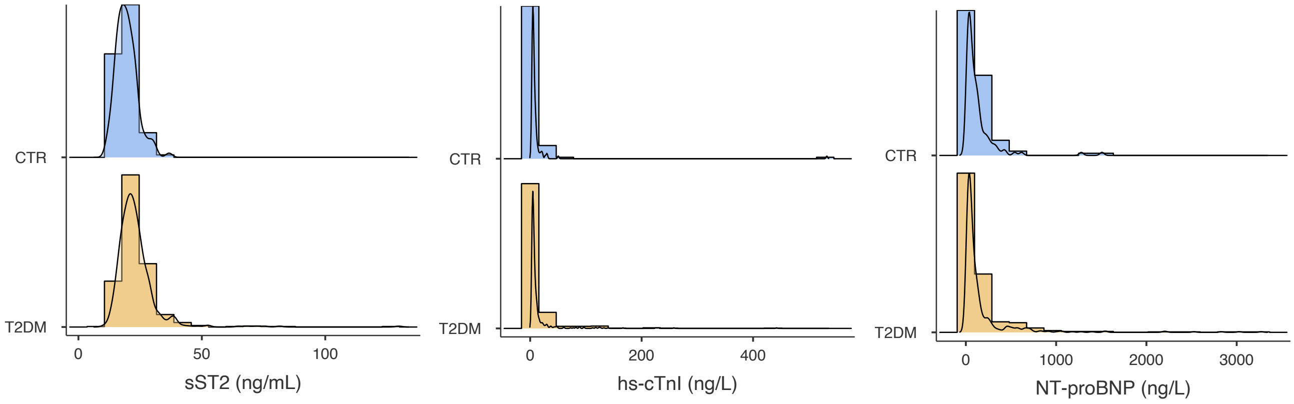

Supplementary Figure 3. (A) Histogram showing the distribution of raw Architect hs-cTnI serum levels among patients with type 2 diabetes (T2DM). **(B)** Passing-Bablok regression between the Siemens Dimension Vista TNIH and the Abbott Architect i-STAT assays for the measurement of hs-cTnI. Regression line with 95% CI is displayed in blue. Line of identity is in red. **(C-D)** Bland-Altman plots showing differences between Dimension Vista TNIH and Abbott's Architect i-STAT hs-cTnI assays for all samples **(C)**, and for mean hs-cTnI values <40 ng/L **(D)**. Lower and upper levels of agreement with 95% CI are displayed in red and green, respectively.

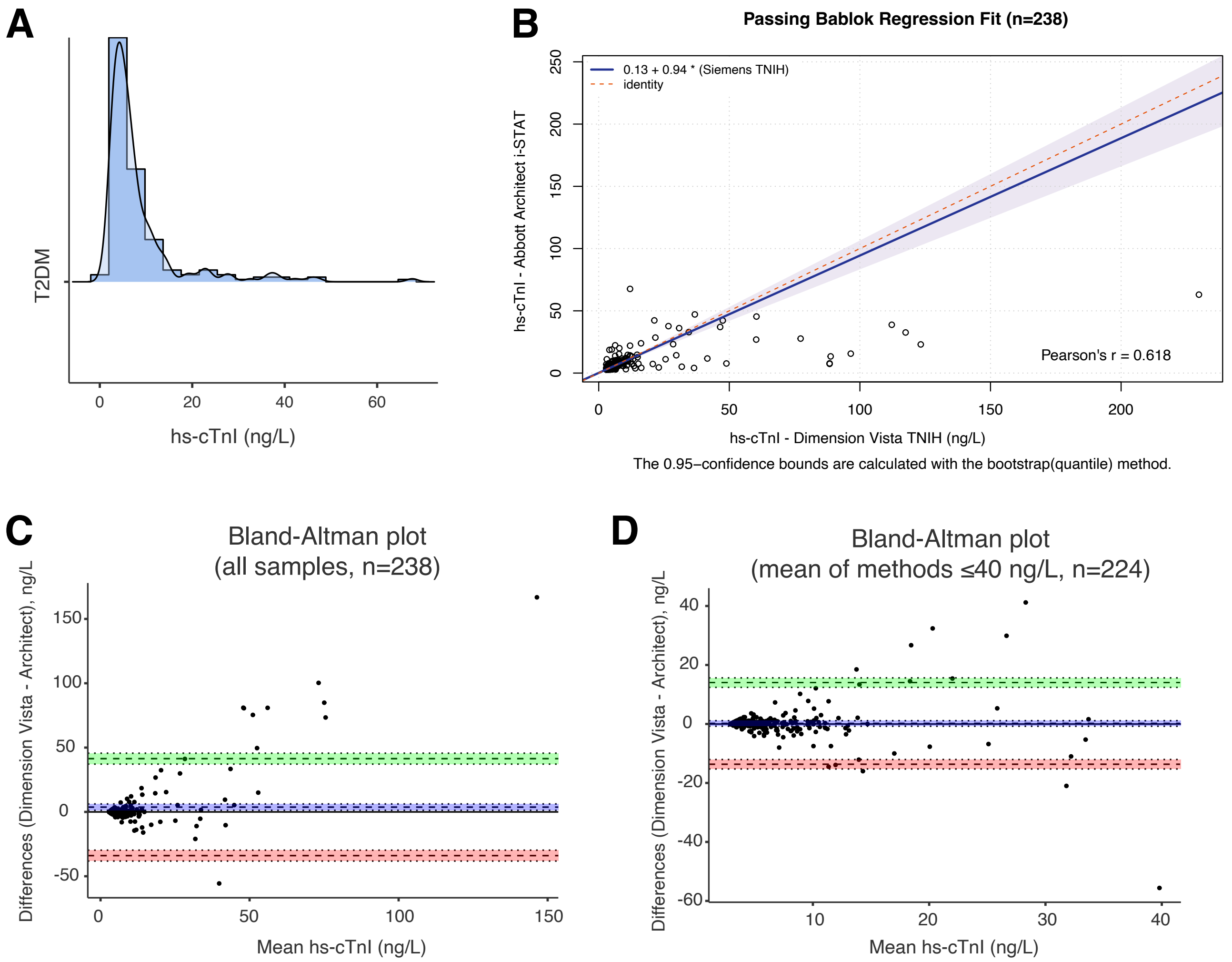

Supplementary Figure 4. (A) Performance of the model based on the internal validation. Model area under the curves (AUCs) at each year are displayed. The solid line represents the mean of the AUC, the dashed line represents the median of the AUC. The darker interval in the plot shows the 25% and 75% quantiles of AUC, the lighter interval shows the minimum and maximum of AUC. **(B)** Internal ed external calibration plots showing the observed against the predicted 15-year survival probability for patients with type 2 diabetes grouped according to risk quartiles. The grey diagonal line represents perfect calibration.

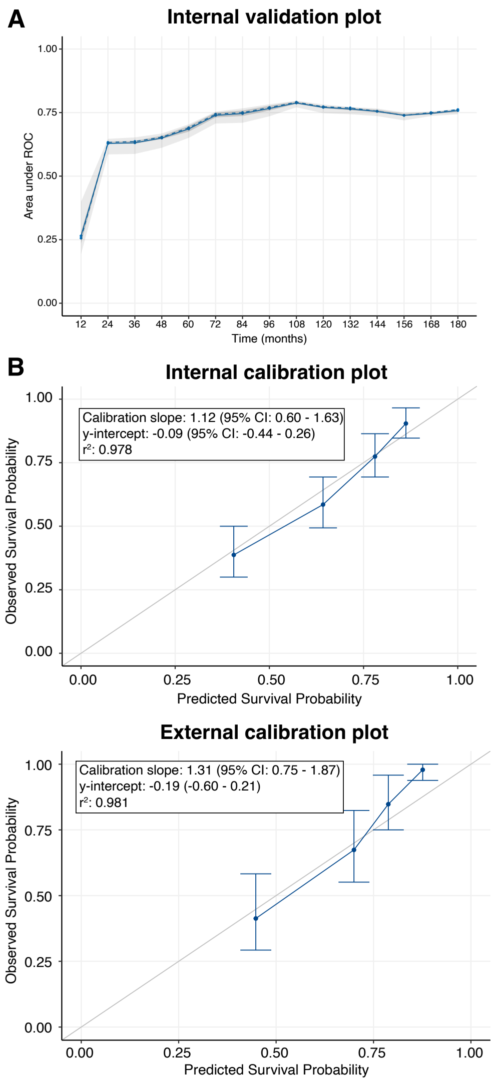

Supplement: Supplementary file 1 — Additional file 1: Table S1. Comparison of raw and log-transformed sST2, hs-cTnI, and NT-proBNP serum levels between healthy control subjects (CTR) and patients with type 2 diabetes mellitus (T2DM). Data are median (IQR). P-values for Mann-Whitney U test. Table S2. Correlation matrix between selected clinical/biochemical variables and serum sST2, hs-cTnI, and NT-proBNP in CTR (n=115) and T2DM (n=568) subjects. Table S3. Results of the MANCOVA model in which log-transformed concentrations of sST2, NT-proBNP, and hs-cTnI were used as dependent variables and each T2DM complication as factor. Age, sex, and HbA1c were used as covariates. Univariate tests for post-hoc comparisons are reported. Arrows indicate significant increase of the dependent variable with complications/treatments. Table S4. C-statistics, with 95% confidence intervals, of the Cox regression models for predicting all-cause mortality in T2DM patients. Table S5. Logistic regression model predicting likelihood of developing the composite endpoint death or MACE in T2DM patients without previous history of MACE. Model summary, χ²=107, df=12, p<0.001, Nagelkerke’s R2=0.284. Table S6. Summary of multiple Cox regression analysis for Architect hs-cTnI levels, categorized according to the best cutoffs (4.4 ng/L and 7.5 ng/L) and to the cardiovascular risk categories defined by the manufacturer, for the prediction of survival in T2DM patients. Crude, adjusted (for sex, age, smoking status, hypertension, T2DM duration, BMI, HbA1c, blood lipids, eGFR, and hs-CRP), and multimarker hazard ratios (HR) with 95% confidence intervals are shown. Significant predictors are in bold Figure S1. Kaplan-Meier survival estimates with 95% confidence intervals for patients with T2DM grouped according to the absence (No) or presence (Yes) of T2DM complications. Figure S2. Distribution of serum sST2, Dimension Vista hs-cTnI, and NT-proBNP serum among healthy controls (CTR) and patients with type 2 diabetes (T2DM). Figure S3. A Histo [file 12933_2022_1616_MOESM1_ESM.pdf]
